# Interpretation of SNP combination effects on schizophrenia etiology based on stepwise deep learning with multi-precision data

**DOI:** 10.1093/bfgp/elad041

**Published:** 2023-09-21

**Authors:** Yousang Jo, Maree J Webster, Sanghyeon Kim, Doheon Lee

**Affiliations:** Department of Bio and Brain Engineering, KAIST, Daejeon, South Korea; Brain Research Laboratory, Stanley Medical Research Institute, Rockville, MD, USA; Brain Research Laboratory, Stanley Medical Research Institute, Rockville, MD, USA; Department of Bio and Brain Engineering, KAIST, Daejeon, South Korea

**Keywords:** SNP combination, GWAS, interpretable machine learning, schizophrenia

## Abstract

Schizophrenia genome-wide association studies (GWAS) have reported many genomic risk loci, but it is unclear how they affect schizophrenia susceptibility through interactions of multiple SNPs. We propose a stepwise deep learning technique with multi-precision data (SLEM) to explore the SNP combination effects on schizophrenia through intermediate molecular and cellular functions. The SLEM technique utilizes two levels of precision data for learning. It constructs initial backbone networks with more precise but small amount of multilevel assay data. Then, it learns strengths of intermediate interactions with the less precise but massive amount of GWAS data. The learned networks facilitate identifying effective SNP interactions from the intractably large space of all possible SNP combinations. We have shown that the extracted SNP combinations show higher accuracy than any single SNPs and preserve the accuracy in an independent dataset. The learned networks also provide interpretations of molecular and cellular interactions of SNP combinations toward schizophrenia etiology.

## INTRODUCTION

Schizophrenia (SCZ) is a serious psychiatric disease characterized by various psychotic episodes. The most common symptoms of schizophrenia are illusions, hallucinations and disorganized thinking [1]. Due to its symptoms, patients fail to recognize what is real and it leads them to unwanted dangerous behaviors such as suicide and crimes. Though there have been many efforts to understand the pathology of schizophrenia, only synaptic and macro-scale natures have been discovered, and its etiology is poorly understood. It has been shown that both environmental and genetic factors are associated with schizophrenia by recent studies. These studies report that maternal risks during pregnancy such as infection and nutritional deficiencies can contribute as environmental factors, and the polygenic effect of many genetic variants can involve as genetic factors [1]. A recent study discovered schizophrenia risk in 31 524 Danish twins and it estimated that the heritability of schizophrenia is 79% [2]. It reveals that schizophrenia is a highly heritable disease and it also supports that finding genetic factors is essential to understand schizophrenia.

To find genetic risk factors of schizophrenia, many groups performed genome-wide association studies (GWAS) using various genotyping methods and cohorts. Psychiatric Genomics Consortium (PGC) collected these GWAS data including over 36 000 cases and 110 000 controls and performed meta-GWAS analysis using these data [3]. From this study, PGC found 108 schizophrenia risk loci. Also, a group in UK integrated PGC data and UK schizophrenia samples and they found a total of 145 schizophrenia risk loci from meta-GWAS analysis [4]. However, it is hard to predict schizophrenia susceptibility with these risk loci because their effect sizes are small due to their polygenic nature. In a polygenic disease including schizophrenia, it is highly demanding to identify multi-SNP effects to find reliable susceptibility markers and understand the pathology [5].

There have been many studies to understand multi-SNP effects but they faced two challenges: exponentially huge search space and limited biological interpretability. Conventional GWAS studies cover 10^5^ ~ 10^6^ SNPs so there are 10^10^ ~ 10^12^ pairs even if we consider only pairs of SNPs. In the computational aspect, the identification of effective multiple SNPs fall into an unmanageable combinatorial search problem. Early studies employed brute-force approaches such as multifactor dimensionality reduction or heuristic-based greedy search algorithms [6, 7]. These methods suffer from the trade-off between search breadth and depth. The other challenge, limited biological interpretability comes from the statistical nature of the GWAS principle itself. Recent studies adopted discriminative pattern mining or differential evolution algorithm to avoid the search space problem and they found significant higher-order multi-SNP effects from large genome-wide datasets [8, 9]. However, these studies provided only ‘statistical’ multi-SNP effects like all other previous studies. They did not provide how SNPs interact with each other or how multi-SNPs affect the phenotype. Therefore, the knowledge underlying multi-SNP effects is still a closed book.

Recently, researchers have tried to apply machine learning to discover underlying knowledge between genotypes and phenotypes by using a concept of visible neural network (VNN) [10]. The authors constructed the architecture of the VNN model with GO ontology and mapped biological roles. Then, the model was trained with a public compendium of yeast growth experimental data. The trained VNN model not only reproduces the output of the system but also provides mediators between genotypes and phenotypes. Though this study shows that deep learning could provide interpretability as well as a prediction in the genotype–phenotype analysis, it cannot be applied to the schizophrenia etiology analysis where a comprehensive and reliable knowledge framework such as GO ontology is not available. After this study, there have been a few efforts to construct interpretable neural network model for human diseases [11–13]. They suggested interpretable models for human diseases but have limitations. High performance models were partially black-box so they are not fully interpretable. Also, these methods only included SNP-gene and gene regulatory interactions so they cannot reflect multilevel nature of biological processes. To overcome this, we chose another approach that utilizes high-precision experiment data for initializing the backbone architecture of deep learning.

In this study, we propose a stepwise deep learning technique with multi-precision data (SLEM) to discover multi-SNP effects of schizophrenia etiology and interpret these effects. SLEM provides an interpretable neural network for schizophrenia in two steps: determination of model architecture using precise multilevel data, and training the architecture using large public GWAS data. Then, we reduced the search space for multi-SNP effects by utilizing information from the acquired SLEM model. Identified SNP combinations can predict schizophrenia patients much better than any single SNPs. Their prediction powers are preserved even if the dataset is changed. We suggest a mechanistic model which could explain how multiple SNPs affect schizophrenia by interpreting the SLEM model. It suggests that four regulatory SNPs constitute a cooperative module to alter the neural cell growth by inducing multiple biological processes including CaMK and BDNF-TrkB pathways.

## METHODS AND MATERIALS

### Overview of SLEM

**Figure 1:**
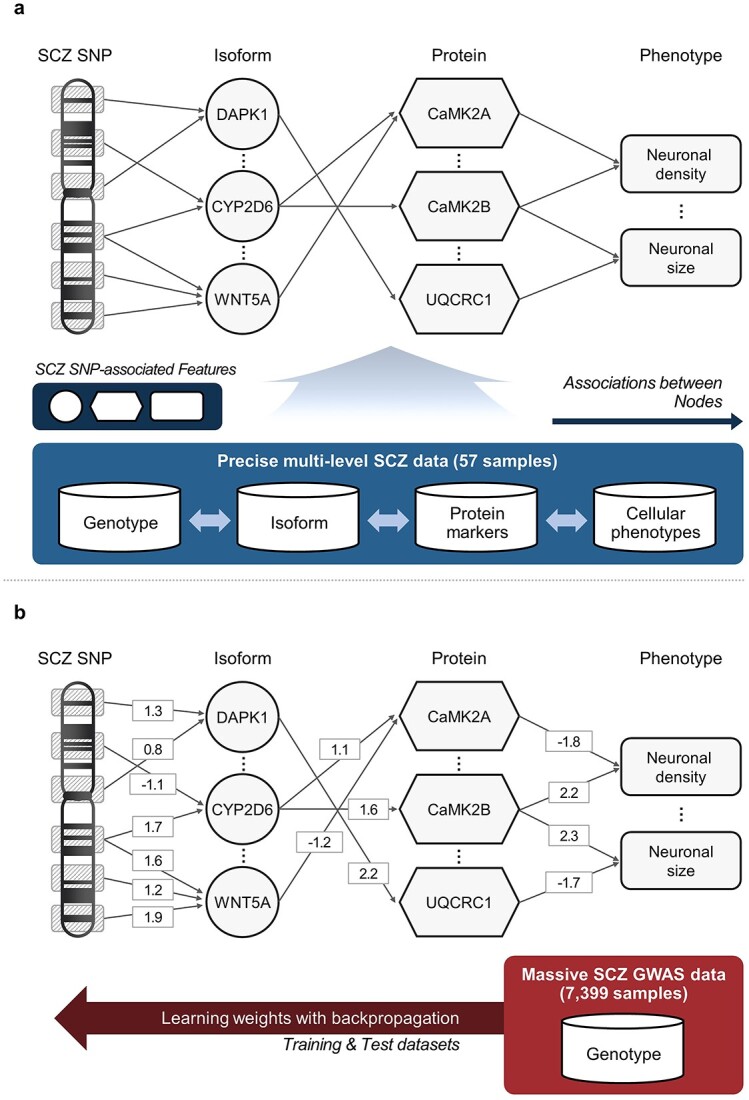
Overview of SLEM. The SLEM trains the interpretable neural network model from multi-precision datasets, precise multilevel data and large end-to-end data. SLEM consists of two steps. (**A**) Determination scheme of network architecture. Nodes for each intermediate layer are selected from precise multilevel SCZ data. Schizophrenia risk SNPs from previous meta-GWAS analysis are selected as input nodes. Transcript isoforms, protein markers and cellular phenotypes having significant association to risk SNPs compose the 1st, 2nd and 3rd intermediate layers, respectively. The initial connectivity between edges is determined by the association significance (*P*-value) between nodes in the adjacent layers. Only significant node pairs have connections and the others are discarded. (**B**) Training procedure of the neural network with large end-to-end data. After determining architecture of the model, the model is trained by the backpropagation algorithm with training and test sets from large end-to-end GWAS datasets.

The proposed SLEM technique consists of two steps: the determination of network architecture and the training (Figure 1). First, nodes for each layer were selected from precise multilevel data (Figure 1A). It is assumed that the precise multilevel data are reflecting the scope and domain of the study. We calculated association scores between schizophrenia risk SNPs and each multilevel feature, and only significantly associated features were chosen as nodes. The connectivity between nodes is determined based on pairwise associations between the adjacent layers (Figure 1A). Only statistically significant connections remain and the other connections are discarded. After the model is initialized, the model proceeds into the training step using the backpropagation algorithm with over 7300 end-to-end (genotype–phenotype) schizophrenia GWAS samples in dbGaP (Figure 1B) [14]. The large training dataset is divided into training and test sets to evaluate the performance of SLEM model by 10-fold cross validation.

### Architecture of interpretable neural networks with SLEM

Interpretable neural networks with the proposed SLEM technique have five layers including an input and a single-node output layer. We selected 138 schizophrenia risk SNPs from previous meta-GWAS analysis and used them as input nodes of the model. In the previous article [4], there are 145 schizophrenia risk loci including findings from PGC [3] but we selected 138 representative SNPs and discarded 7 indel loci, which show low accuracy in variant calling. Also, we determined that the former layers simulate micro-scale processes and the latter layers reflect macro-scale processes because biological signals are sent from micro-scale to macro-scale processes. Considering data availability, we set three intermediate layers for transcript isoforms, protein markers and cellular phenotypes, respectively. We used transcript isoforms instead of genes because a recent study reported that isoform-specific expression has an essential role in schizophrenia pathology [15].

### Stanley Medical Research Institute dataset for the first step of SLEM

We employed Neuropathology consortium (NC) collection of the Stanley Neuropathology Consortium Integrative Database (SNCID) for the first step of SLEM [16]. The NC collection consists of postmortem brain samples of 15 each diagnosed with schizophrenia, bipolar disorder or major depression and unaffected control. For each sample, there are whole genome sequencing (WGS) data, multi-tissue RNA-sequencing data (RNA-seq) and neuropathological experiments data including over 4000 traits. We focused on frontal cortex, which has the richest schizophrenia omics data in SNCID and has known to be closely related to schizophrenia. Among 60 samples, we selected 57 samples that have all three types of data. We treated them as 15 cases versus 42 controls for tasks for finding associations with schizophrenia.

We extracted the genotype profile from WGS data. For each sample, raw genome sequence data (FASTQ) is aligned to the GRCh37 reference genome by using the HISAT2 aligner [17]. Schizophrenia risk loci profile is called from sequence aligned map by the BCFtools software [18]. The profile of 138 risk SNPs is gathered for all 57 samples from this procedure. We analyzed the isoform expression profile from RNA-seq data. We quantified the isoform-level expression of every sample by using the Salmon software [19]. Among over 190 000 isoforms in GRCh37, we removed low expressed isoforms and selected 57 404 isoforms. We gathered protein marker and cellular phenotype profiles from neuropathological experiment data. We removed experiments with too many missing values (>25% missing) and prepared 887 experimental features for every sample.

### Selection of intermediate nodes based on associations with risk SNPs

There are three intermediate layers for isoforms, protein markers and cellular phenotypes in the SLEM model. Each layer should have nodes that represent the essential components of schizophrenia etiology. We performed an eQTL analysis to find significant isoforms for the 1st layer. We prepared the schizophrenia risk SNP profile and the isoform expression profile from the SNCID dataset. We extracted significant SNP-isoform associations using the linear model in the QTLtools software [20]. We adjusted eQTL associations with age, sex, disease profile and 15 PEER factors (hidden confounders in expression data) [21]. From SNP-isoform associations, we selected isoforms that have FDR adjusted *P*-value < 0.1. In eQTL analysis, we tested over 9000 000 times (138 risk SNPs versus >70 000 transcripts) with only 57 samples, so the statistical significance of associations is relatively low, so we chose the threshold as 0.1, instead of 0.05. As a result, we found 67 significant isoforms associated with schizophrenia risk SNPs and used them as nodes of the 1st layer.

We also performed statistical association studies to find significant protein markers and cellular phenotypes for the 2nd and 3rd layers. From schizophrenia risk SNP profile and neuropathological experiment data, we identified significant SNP-protein marker and SNP-cellular phenotype associations using a generalized linear model in the plink2 software [22]. Then, we adjusted associations with age, sex and disease profile. We selected markers and phenotypes that have FDR adjusted *P*-value < 0.05 and identified 42 significant protein markers and 19 significant cellular phenotypes associated with schizophrenia risk SNPs.

### Determination of the connectivity of edges between layers

To determine the connectivity between layers, we extracted pairwise associations between nodes in adjacent layers and computed initial weights for the training based on their statistical significance (*P*-value). For each node pair from two adjacent layers, we computed the *P*-value between nodes and converted it into its corresponding weight from Xavier normal distribution, which is widely used for neural network initialization. And the sign of the weight is determined based on whether the association is positive or negative (Supplementary Figure S1).

For SNP-isoform edges, we used nominal *P*-value from eQTL analysis of the preceding section as statistical significance. We computed nominal *P*-values for all pairs between 138 input risk SNPs and 67 isoform nodes. Then, we converted these *P*-values into Xavier-distributed weights by inverse transform and selected positive or negative weights based on the slope value of the eQTL linear model. For isoform-marker and marker-phenotype edges, we performed the robust nonparametric correlation test, Kendall rank correlation test [23] to find associations and their statistical significances because many features in the neuropathological experiment dataset are not normally distributed. Then, *P*-values are transformed into Xavier-distributed weights and signs of weights are determined by signs of Kendall coefficients. Also, the phenotype-schizophrenia edges are initialized with Xavier normal distribution.

From these initial weights, only the top 35% of edges (based on absolute value) in each layer are used for the training step and the rest of the edges are masked as 0 and discarded because weak associations may affect the training procedure and lead the model to same optima with randomly initialized models if they are not properly masked. A total of 19 edges between phenotypes and schizophrenia are initialized by Xavier initialization because they do not have initial weight information.

### Preparation of training and test datasets for the second step of SLEM

We prepared three dbGaP schizophrenia datasets11 (phs000021.v3.p2, phs000167.v1.p1, phs000448.v1.p1) for SLEM training and the evaluation. These datasets are end-to-end datasets that only contain genotype data as features and schizophrenia diagnosis as a label. Unlike the SNCID dataset, genotype data in dbGaP datasets are SNP array data so datasets cover only ~10% of selected schizophrenia risk SNPs. To solve this problem, we performed the SNP imputation procedure to dbGaP SNP array datasets.

The SNP imputation procedure is the pipeline to extract genotypes of unknown SNPs based on partially known genotypes and haplotype information. First, we converted genomic coordinates of datasets from GRCh36 into GRCh37 by using LiftOver software [24]. Then, we inferred haplotypes of every sample using SHAPEIT phasing software and 1000 Genome Project haplotype reference [25, 26]. Lastly, we extracted the genotype profile of 138 schizophrenia risk SNPs from prephased haplotype information using the IMPUTEv2 software [27]. Extracted genotypes are coded into 0 (homozygous reference allele), 1 (heterozygous), 2 (homozygous alternative allele) based on SNP additive model assumption.

### Model implementation and training

We implemented the SLEM model using the Keras library [28]. The model receives 138 genotype profiles as vectors and predicts whether each sample is schizophrenia patients or not by using a sigmoid classifier in the last layer. Also, we constructed the model to predict the on–off status of 67 isoforms, 42 protein markers and 19 cellular phenotypes applying values from the previous layer to the hyperbolic tangent activation function. Edges between layers are initialized by initial weights from the preceding section and insignificant edges are masked in the training step.

We trained the SLEM model with the dbGaP schizophrenia datasets [14] using the backpropagation algorithm with the ADAM optimizer [29]. To find optimal training hyperparameters, we evaluated accuracies from 10-fold cross-validation varying learning rate, batch size and the number of epochs. From hyperparameter tuning, we determined 0.005 learning rate, 250 batch size and 750 epochs as optimal hyperparameters and trained and evaluated the SLEM model with these hyperparameters.

To examine the reliability of the SLEM model, we compared the overall accuracy of the SLEM model and a conventional neural network model with fully connected edges. The latter has the same number of layers and nodes as the SLEM model, but has 12 877 edges for full connection. Each node in the fully connected model plays a role of computational transfer function without any biological label. Note that the proposed SLEM model has only 1192 edges after aforementioned selections, and each node has an explicit biological label.

## RESULTS

### Prediction accuracy evaluation for model reliability

We measured the average AUROC of 100 trained models for each of both models. The AUROC of the SLEM model marks 0.7502 and that of the fully connected model is 0.7348. The result shows that the SLEM model has higher performance than the fully connected network. It implies that the SLEM model has acceptable amount of information contents and organized structures to discriminate schizophrenia patients, and maintains biological interpretability.

### A suggested mediator pathway module for neuronal cell growth

**Figure 2:**
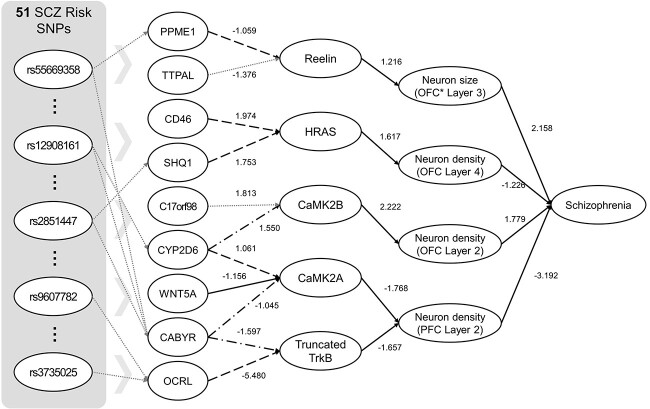
A neuronal growth model discovered from SLEM and literature validation of each association. Numbers around edges are weights of associations. We searched and found literature evidence of each edge (solid line), database annotation (dashed line), and protein domain interaction (dash-dotted line).

We extracted a mediator pathway module for neuronal cell growth by projecting the whole trained SLEM network into neuronal cell growth phenotypes, which has been known to be an essential biological aspect of schizophrenia etiology (Figure 2). To extract mediator pathways, we left only top 10% absolute weights in trained SLEM model because there are too many weak edges. Then, we collected all nodes and edges that are connected to neuronal cell phenotypes (neuronal cell size and density).

In our literature survey, we found three genes in the isoform layer of models have schizophrenia-related reports (cytochrome P450 2D6 (CYP2D6), WNT5A, CD46). Four genes or proteins in our models (calcium/calmodulin dependent protein kinase 2 (CaMK2), tropomyosin receptor kinase B (TrkB), WNT5A, HRAS) were reported to play essential roles in BDNF-TrkB and WNT signaling pathways that modulate neuronal growth, neuronal proliferation and synaptic plasticity (Supplementary Table S1) [30–32].

We have found that 90% of edges in the suggested module have their supporting evidences from independent information sources (Figure 2). We examined literature and database annotations including GO, KEGG and Reactome. We expanded the search space of protein–protein interactions (PPI) to structural family proteins (Pfam), which includes domain-level interactions. Considering insufficient knowledge status on schizophrenia etiology, it is inspiring that 90% of edges in the machine-learned models have supporting evidences from independent knowledge sources (Supplementary Table S2). We expect that the mediator pathway module by SLEM illustrate the neurodevelopmental aspect of schizophrenia in a mechanistic way.

### Identification of SNP combinations from the SLEM model

We evaluated an individual impact score of each SNP on the SLEM model. In each node, we assumed that active inputs are combined in additive manner and the output is proportional to output weight w_o_. We defined relationship between active inputs (i_1,_ …, i_n_) and the output of a node k (O_k_) as the following equation:


(1) \begin{equation*} {\mathrm{O}}_k={\mathrm{w}}_k\sum_1^n{i}_j \end{equation*}

**Figure 3:**
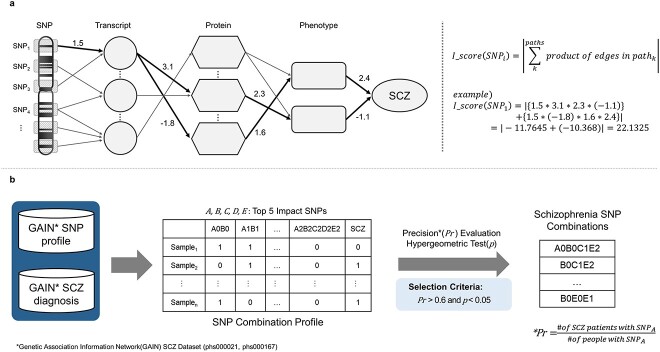
Selection of high impact SNP combinations from the SLEM model. (**A**) An individual impact score of each SNP is defined as the sum of weights in all possible paths from the SNP to schizophrenia. (**B**) Effective SNP combinations are selected from all possible combinations from the top five high impact SNPs. SNP combinations with high precision (precision > 0.6) with statistical significance (*P*-value < 0.05) on all samples in the GAIN dataset are chosen as effective schizophrenia SNP combinations. In the table, A, B, C, D and E refer to labels of the five highest impact SNPs in the model, and 0, 1 and 2 indicate homozygous reference, heterozygous and homozygous alternative allele, respectively. For example, A0B1C2 is a combination that SNP A is homozygous reference, SNP B is heterozygous and SNP C is homozygous alternative in one sample.

The impact score of each SNP is defined as the sum of inputs to schizophrenia node and it can be represented by iteration of Equation (1). Since Equation (1) is linear combination so the impact score is equivalent to Equation (2) that the sum of products of edges in all possible path from the SNP to schizophrenia (Figure 3A). We used the absolute value to evaluate the magnitude of impact.


(2) \begin{equation*} I\_ score\left( SN{P}_i\right)=\left|{\sum}_k^{paths} product\ of\ edges\ in\ pat{h}_k\right| \end{equation*}

Based on the impact score, we selected the five highest impact SNPs (Supplementary Table S3). Then, we selected SNP combinations by evaluating the prediction power of every possible combination of the five highest impact SNPs (Figure 3B). Since an SNP combination is a far stricter condition than a single SNP, it is straightforward that the recall of an SNP combination is lower than that of a single SNP. Therefore, we measured the precision that each SNP combination discriminates schizophrenia patients in the public GWAS dataset [Genetic Association Information Network (GAIN) dataset: phs000021.v3.p2, phs000167.v1.p1]. We also performed hypergeometric test to check statistical significance of predictions. We used all single SNPs used for SLEM as a control group and measured precisions (the indicator of prediction power; a number of patients with a marker / a number of all people who have a marker) and hypergeometric *P*-values. As reliable schizophrenia SNP combinations, we selected SNP combinations that satisfy high prediction power (precision > 0.6) with statistical significance (*P*-value < 0.05) (Table 1). From these criteria, we can consider that not all schizophrenia patients have these SNP combinations but people who have these combinations may have high schizophrenia susceptibility and also these multi-SNP effects can be found regardless of the dataset variation. It implies that the SLEM model properly reflects the polygenic nature of schizophrenia and provides powerful indicators of schizophrenia.

**Table 1:** Schizophrenia SNP combinations and single SNPs satisfying criteria (precision > 0.6 and hypergeometric *P*-value < 0.05)

**Model**	**SNP**	**Precision** **(GAIN)**	**Precision** **(HUGR)**	*P* **-value** **(GAIN)**	*P* **-value** **(HUGR)**
Best single SNPs	rs344252	0.541 (761/1407)	0.289 (30/104)	0.001	de-enriched
	rs12207258	0.529 (699/1322)	0.31 (100/323)	0.008	de-enriched
	rs6811243	0.523 (559/1069)	0.315 (141/449)	0.041	de-enriched
	rs56145559	0.519 (1987/3835)	0.322 (263/819)	0.001	de-enriched
	rs1319017	0.516 (2600/5042)	0.291 (509/1753)	0.001	de-enriched
	rs6002655	0.514 (2816/5486)	0.313 (604/1934)	0.001	de-enriched
	rs489939	0.512 (1312/2567)	0.309 (528/1710)	0.05	de-enriched
	rs7701440	0.508 (3063/6032)	0.304 (602/1984)	0.001	de-enriched
	rs783540	0.507 (2368/4676)	0.313 (570/1825)	0.028	de-enriched
Neuronal growth model	A2D0	0.769 (10/13)	0.667 (2/3)	0.044	0.5
	A0B1C2	0.618 (34/55)	1 (2/2)	0.048	0.25
	A1D0E2	0.818 (9/11)	0.5 (1/2)	0.032	0.75
	A2D0E0	0.875 (7/8)	0.667 (2/3)	0.034	0.5
	B1C2D0	0.643 (27/42)	1 (1/1)	0.041	0.5
	B1C2E0	0.66 (31/47)	1 (1/1)	0.018	0.5
	C0D0E2	0.673 (33/49)	0.5 (7/14)	0.01	0.605
	A0B1C2D0	0.676 (25/37)	1 (1/1)	0.022	0.5
	A0B1C2E0	0.651 (28/43)	1 (1/1)	0.031	0.5
	A1B0D0E2	0.818 (9/11)	0.5 (1/2)	0.032	0.75
	A2B0D0E0	1 (6/6)	0.667 (2/3)	0.015	0.5
	A0C0D0E2	0.636 (28/44)	0.5 (6/12)	0.045	0.613
	B0C0D0E2	0.659 (29/44)	0.545 (6/11)	0.022	0.5
	B1C2D0E0	0.667 (20/30)	1 (1/1)	0.047	0.5
	A0B1C2D0E0	0.679 (19/28)	1 (1/1)	0.041	0.5

Numbers next to precision refer numbers of true positives (patients with a marker) and positives. De-enriched in *P*-value indicates that a marker discriminates patients less than random (50%). In each combination, A, B, C, D and E denote labels of the five highest impact SNPs in the model (Table S1), and 0, 1 and 2 indicate homozygous reference, heterozygous and homozygous alternative allele, respectively. For example, A0B1C2 is a combination that SNP A is homozygous reference, SNP B is heterozygous and SNP C is homozygous alternative in one sample.

**Figure 4:**
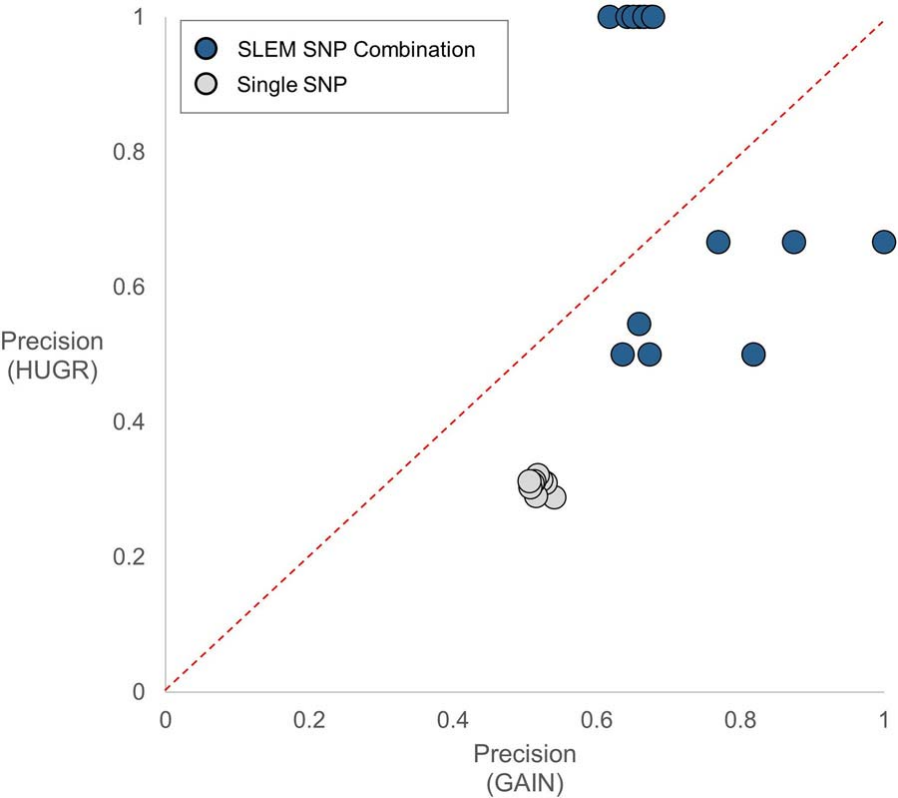
Scatter plot comparison of SNP combinations and single GWAS SNPs. Each dot represents an SNP combination or a single SNP in Table 1. Single SNPs (gray) are biased toward the lower side and it shows that single SNPs lose their prediction power in the independent dataset. In contrary to single SNPs, SNP combinations from the SLEM model (blue) are close to the identity line. It indicates that SNP combinations from SLEM preserve prediction power in different datasets and they are more robust schizophrenia markers than single GWAS SNP.

We evaluated performances in independent test dataset [Hebrew University Genetic Resource (HUGR) dataset: phs000448.v1.p1] to compare performances of SNP combinations and single SNPs. The SNP combinations from SLEM models show higher and more robust performance than single GWAS SNPs. The single SNPs that have the highest precision show 0.545 in the GAIN training dataset and 0.375 HUGR test datasets (Table 1). Also, the minimum precision difference between the two datasets is 0.140. It implies that even a confirmed single SNP from the meta-GWAS analysis cannot discriminate schizophrenia patients by itself and also cannot guarantee robustness in independent datasets. On the contrary, the best SNP combination shows over 60% precisions in both datasets. The scatter plot visualizes this difference between single SNPs and SNP combinations (Figure 4). Single SNPs are located on the lower side of the figure and it indicates that they have low precisions in HUGR datasets. However, SNP combinations are close to the identity line in the middle and it implies that they have similar precisions in both datasets. It clearly illustrates performance and robustness of SNP combinations as schizophrenia markers.

### Suggested multi-SNP effects on schizophrenia

**Figure 5:**
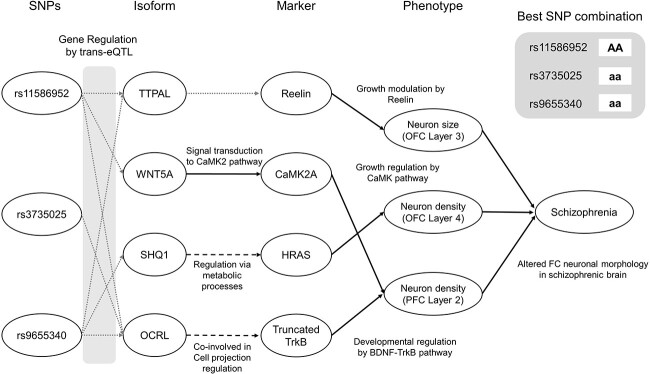
Identified SNP combinations along with their intermediate effect pathways to schizophrenia susceptibility. Each association is explained by potential biological interactions developed from literature evidences (Figure 2). The SNP combination with the best performance is selected as the most reliable combination from each model. Allele notations ‘aa’, ‘AA’ and ‘Aa’ refer to homozygous reference, homozygous alternative and heterozygous, respectively.

We suggest multi-SNP combinational effects on schizophrenia depicted in Figure 5. The combination model is extracted from the whole SLEM networks by selecting connecting paths originated from the most effective SNP combination, which is selected from the aforementioned screening. The machine-learned connections in the suggested model provide guidance to examine the corresponding literature annotations.

We found that two SNPs (rs11576952 and rs9655340) are located in the noncoding regions so they are distant from exons. Thus, they have trans-eQTL associations with isoforms so they may regulate mRNA expressions through intermediary factors. Remaining one, rs3735025, is located in exon of gene diacylglycerol kinase iota (DGKI). DGKI and connected gene OCRL are both involved in Phosphatidylinositol signaling system (KEGG:04070) so rs3735025 may affect OCRL by lipid modification signaling via DGKI. The mRNA expressions are determined by combined regulatory effects of all three SNPs. In the isoform layer, isoforms may affect their partners in two ways. The first way is indirect interactions from co-involved metabolic processes (green line). For example, OCRL and TrkB are co-involved in the cell projection regulation process, so we can expect that OCRL expression may change TrkB expression through intermediary genes or proteins in cell projection regulation process. The other way is WNT-Ca2+ signaling (blue line), which is well known by literatures [32]. Unfortunately, there is no report about the interaction between Alpha Tocopherol Transfer Protein Like (TTPAL) and Reelin but it may be derived from the lack of knowledge of TTPAL itself (only six reports in pubmed). In the marker layer, protein Reelin, HRAS and CaMK2A may regulate neuronal growth by downstream effectors and CaMK pathway [33–37] and TrkB would contribute to neuronal development by BDNF–TrkB pathways [30–32]. As a consequence, neuronal size and density in the frontal cortex would be altered like already reported in previous studies [38].

## DISCUSSION

We have proposed SLEM to explore the effect of SNP combinations on the cause of schizophrenia through intermediate molecular and cellular functions. The proposed SLEM technique utilizes two levels of precision data for learning. It constructs initial backbone networks with more precise but small amount of multilevel assay data. Then, it learns relative weights of intermediate interactions with the less precise but massive amount of public GWAS data. The learned networks facilitate identifying effective SNP interactions from the intractably large space of all possible SNP combinations.

We have identified 15 effective SNP combinations including a combination of rs11586952(AA), rs3735025(aa) and rs9655340(aa) along with their intermediate effect pathways to schizophrenia susceptibility in the neural growth model (Figure 5 and Table 1). We expect that these combinations are worthy of further examination to deepen mechanistic understanding of schizophrenia etiology.

In the suggested mediator pathway module, the cellular phenotypes show different associations by neocortical layers. Neuron density of orbitofrontal cortex (OFC) layer 4 has a negative association with schizophrenia, whereas neuron density of OFC layer 2 has a positive association. Typically, it is a rare phenomenon that two close layers have opposite associations with schizophrenia but cell type heterogeneity caused by neuronal migration may explain the case. It is a widely known fact that neurons migrate during the development of the cerebral cortex [39]. And this migration leads to the heterogeneity of cell type composition in cortical layers. Single-cell sequencing unveils cellular migration during cortical development and also reveals cell type heterogeneity in the late stage of the development [40]. This cell type heterogeneity caused by migration results in different cell sizes or densities by layer and opposite associations in SLEM model. Since there are limited neocortical layer-specific studies, it is not simple to verify this assumption but there is a supporting study that abnormal neuronal migration is implicated in schizophrenia [41].

We also expect that the proposed SLEM technique could be applied to other disease studies, where (i) massive GWAS data are available, (ii) small scale but precise assay data are available and (iii) disease pathology in the molecular and cellular contexts is still controversial or vague.

**Key Points**

- We propose an interpretable deep learning technique, which called SLEM to discover multi-SNP effects of schizophrenia etiology and interpret these effects.
- SLEM technique can train the interpretable model for schizophrenia that has insufficient prior information and samples by utilizing information of experimental data.
- We confirmed that the trained SLEM model shows comparable performance to the conventional model and its interpretable information is consistent with previous knowledge.
- We found SNP combinations that predict schizophrenia more precise and robust and discovered their multi-SNP effects on neuronal growth by interpreting information of the SLEM model.

## Supplementary Material

Supplementary_figures_tables_230903_elad041

supplementary_data_S1_elad041

revision_suppl_no1_SLEM_connection_vs_accuracy_elad041

SLEM_BIB_supplementary_230513_elad041

## Data Availability

The data underlying this article are available in the article and in its online supplementary material.

## Author Biographies

**Yousang Jo** is a PhD student at Department of Bio and Brain Engineering in KAIST. He focuses on multi-omics analysis and machine learning.

**Maree J. Webster** is the executive director at Stanley Medical Research Institute. She focuses on studies of psychiatric disorders including schizophrenia and bipolar disorder.

**Sanghyeon Kim** is associate director of database management at Brain Research Laboratory in Stanley Medical Research Institute. He focuses on neuropathology of psychiatric disorder via both data analysis and experiments.

**Doheon Lee** is a professor at Department of Bio and Brain Engineering in KAIST. His research interests include biomedical informatics and data mining.
