# Supplementary material for: Interpretation of SNP combination effects on schizophrenia etiology based on stepwise deep learning with multi-precision data
Source: Brief Funct Genomics. 2023 Sep 21;23(5):663–71. doi: 10.1093/bfgp/elad041 (PMC11428150; doi:10.1093/bfgp/elad041)
Supplement: Supplementary_figures_tables_230903_elad041 [file supplementary_figures_tables_230903_elad041.pdf]

Supplementary Materials for

Interpretation of SNP combination effects on schizophrenia etiology based on stepwise deep learning with multi-precision data

Yousang Jo[1], Maree J. Webster[2], Sanghyeon Kim[2,*] and Doheon Lee[1,*]

*Corresponding E-mail: dhlee@kaist.ac.kr (Doheon Lee)

s:

Figures S1
Tables S1 to S3
Data S1

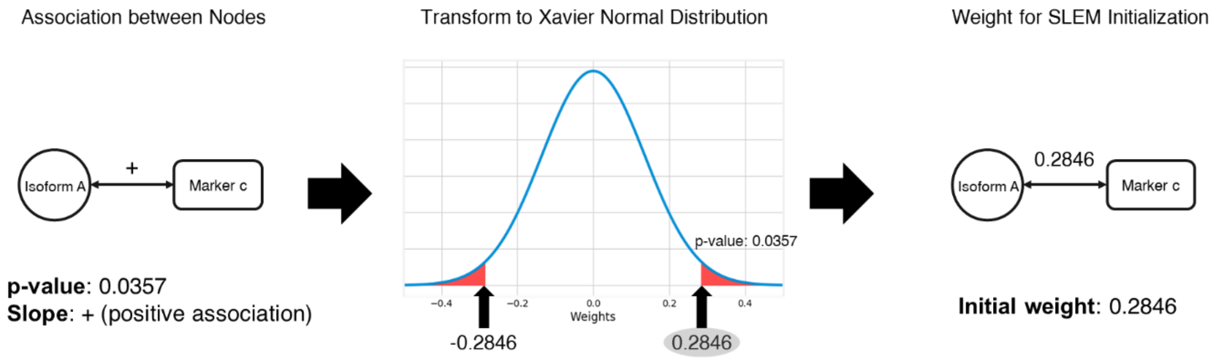

Figure S1. The conversion procedure from an association to corresponding weight for SLEM initialization. Associations between nodes in two layers are found by association studies (eQTL analysis and statistical test). Each association has its statistical significance (p-value) and information about sign (positive or negative). P-value of each association is converted to corresponding weight in Xavier normal distribution by inverse normal transformation. Then, sign of each weight is determined by the sign of original association and converted weights are used for SLEM initialization

Table S1. Nodes in mediator pathway models which have schizophrenia-related reports or related to neurodevelopmental processes. Numbers of citation refer to references in the original article.

Gene	Reported association to schizophrenia
CYP2D6	Reported from multiple schizophrenia GWAS and eQTL analysis [3, 42-43].<br>Variation and expression change contribute to schizophrenia [42-43].
CD46	Complement-control genes are associated to schizophrenia [44].
WNT5A	Differential gene expression of WNT5A pathway genes are reported from schizophrenia patients [45].<br>Crosstalk between BDNF-TrkB and Wnt signaling modulates brain development [30].
CaMK2	CaMK pathway regulates neuronal growth and synaptic plasticity [34-35].
HRAS	Ras signaling plays an important role in BDNF-TrkB signaling [31].
TrkB	BDNF-TrkB pathway regulates various neuronal and glial development processes [30-32].

References for Table S1

42. Ma L, Shcherbina A, Chetty S. Variations and expression features of CYP2D6 contribute to schizophrenia risk. *Mol Psychiatry 2020 266* 2020;**26**:2605–15.

43. Kirchheiner J, Seeringer A, Godoy AL *et al.* CYP2D6 in the brain: genotype effects on resting brain perfusion. *Mol Psychiatry 2011 163* 2010;**16**:333–41.

44. Håvik B, Le Hellard S, Rietschel M *et al.* The Complement Control-Related Genes CSMD1 and CSMD2 Associate to Schizophrenia. *Biol Psychiatry* 2011;**70**:35–42.

45. Evgrafov O V., Armoskus C, Wrobel BB *et al.* Gene Expression in Patient-Derived Neural Progenitors Implicates WNT5A Signaling in the Etiology of Schizophrenia. *Biol Psychiatry* 2020;**88**:236–47.

Table S2. Literature evidences for associations in the mediator pathway models. Numbers of citation refer to references in the original article.

Model	Upstream	Downstream	Reported evidence
Neuronal growth model	Reelin	Neuron size	Reelin regulates neuronal growth [33].<br>Size of soma is decreased in reelin-deficient neurons [36].
	CaMK2A	Neuronal density	CaMK pathway regulates neuronal growth and synaptic plasticity [34-35].
	CaMK2B	Neuronal density	CaMK pathway regulates neuronal growth and synaptic plasticity [34-35].
	WNT5A	CaMK2A	Wnt signaling modulates activation of CaMK family [32].
	TrkB	Neuronal density	BDNF-TrkB pathway regulates various neuronal and glial development processes [30–32].
	HRAS	Neuronal density	Downstream effectors of HRAS regulates survival, growth and differentiation of neurons [37].
	Neuronal density	Schizophrenia	Downward shifts in neuron size and increased small neuron density is reported in schizophrenic prefrontal cortex [38].
	Neuron size		

Table S3. SNPs which have highest impact in the neuronal growth model. Each SNP is labeled as A, B, C, D, and E for readability in SNP combination.

SNP	Label	Impact Score
rs11586952	A	72.816
rs17517490	B	61.181
rs12344647	C	59.337
rs3735025	D	56.415
rs9655340	E	54.580

Data S1. (separate file)

The full list of nodes and edges of the trained SLEM model.
