# Supplementary material for: Interpretation of SNP combination effects on schizophrenia etiology based on stepwise deep learning with multi-precision data
Source: Brief Funct Genomics. 2023 Sep 21;23(5):663–71. doi: 10.1093/bfgp/elad041 (PMC11428150; doi:10.1093/bfgp/elad041)
Supplement: SLEM_BIB_supplementary_230513_elad041 [file slem_bib_supplementary_230513_elad041.docx]

*Supplementary Materials for*

**Interpretation of SNP combination effects on schizophrenia etiology based on stepwise deep learning with multi-precision data**

# Yousang Jo1, Maree J. Webster2, Sanghyeon Kim2,* and Doheon Lee1,*

*Corresponding E-mail: [dhlee@kaist.ac.kr](mailto:dhlee@kaist.ac.kr%20) (Doheon Lee)

**This supplementary material includes:**

Figures S1

Tables S1 to S3

Data S1


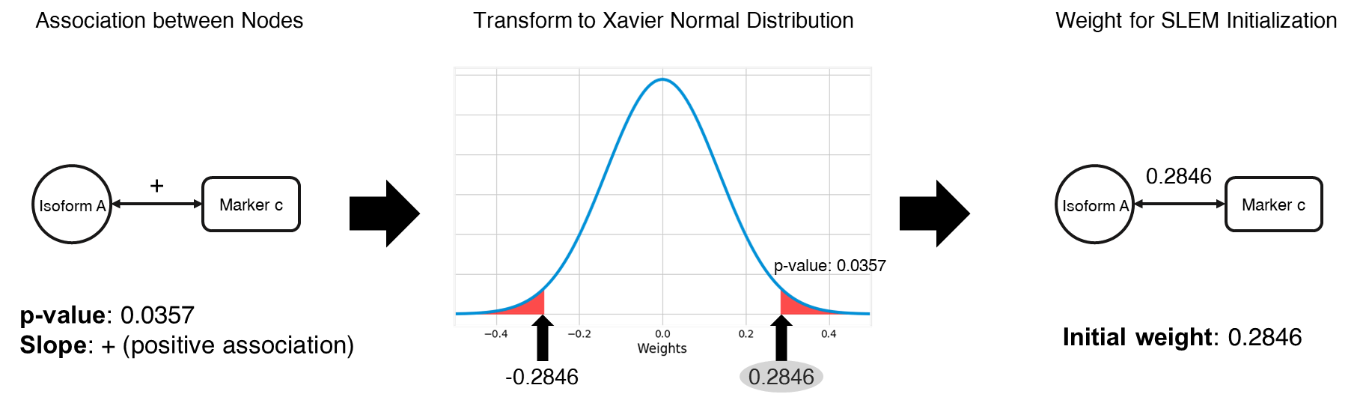


Figure S1. The conversion procedure from an association to corresponding weight for SLEM initialization. Associations between nodes in two layers are found by association studies (eQTL analysis and statistical test). Each association has its statistical significance (p-value) and information about sign (positive or negative). P-value of each association is converted to corresponding weight in Xavier normal distribution by inverse normal transformation. Then, sign of each weight is determined by the sign of original association and converted weights are used for SLEM initialization

Table S1. SNPs which have highest impact in the neuronal growth model. Each SNP is labeled as A, B, C, D, and E for readability in SNP combination.

| SNP | Label | Impact Score |
| --- | --- | --- |
| rs11586952 | A | 72.816 |
| rs17517490 | B | 61.181 |
| rs12344647 | C | 59.337 |
| rs3735025 | D | 56.415 |
| rs9655340 | E | 54.580 |

Table S2. Nodes in mediator pathway models which have schizophrenia-related reports or related to neurodevelopmental processes. Numbers of citation refer to references in the original article.

| Gene | Reported association to schizophrenia |
| --- | --- |
| CYP2D6 | Reported from multiple schizophrenia GWAS and eQTL analysis [3, 42- 43].  Variation and expression change contribute to schizophrenia [42-43]. |
| CD46 | Complement-control genes are associated to schizophrenia [44]. |
| WNT5A | Differential gene expression of WNT5A pathway genes are reported from schizophrenia patients [45].  Crosstalk between BDNF-TrkB and Wnt signaling modulates brain development [30]. |
| CaMK2 | CaMK pathway regulates neuronal growth and synaptic plasticity [34-35]. |
| HRAS | Ras signaling plays an important role in BDNF-TrkB signaling [31]. |
| TrkB | BDNF-TrkB pathway regulates various neuronal and glial development processes [30-32]. |

**Table S3. Literature evidences for associations in the mediator pathway models.** Numbers of citation refer to references in the original article.

| Model | Upstream | Downstream | Reported evidence |
| --- | --- | --- | --- |
| Neuronal growth model | Reelin | Neuron size | Reelin regulates neuronal growth [33].  Size of soma is decreased in reelin-deficient neurons [36]. |
|  | CaMK2A | Neuronal density | CaMK pathway regulates neuronal growth and synaptic plasticity [34-35]. |
|  | CaMK2B | Neuronal density | CaMK pathway regulates neuronal growth and synaptic plasticity [34-35]. |
|  | WNT5A | CaMK2A | Wnt signaling modulates activation of CaMK family [32]. |
|  | TrkB | Neuronal density | BDNF-TrkB pathway regulates various neuronal and glial development processes [30–32]. |
|  | HRAS | Neuronal density | Downstream effectors of HRAS regulates survival, growth and differentiation of neurons [37]. |
|  | Neuronal density | Schizophrenia | Downward shifts in neuron size and increased small neuron density is reported in schizophrenic prefrontal cortex [38]. |
|  | Neuron size |  |  |

Data S1. (separate file)

The full list of nodes and edges of the trained SLEM model.
